# Effect of Fluorometholone/Tetrahydrozoline Fixed Combination on Conjunctival Autograft Morphology after Primary Pterygium Excision

**DOI:** 10.1155/2013/481843

**Published:** 2013-06-20

**Authors:** Engin Bilge Ozgurhan, Necip Kara, Ercument Bozkurt, Baran Gencer, Alper Agca, Zeynep Alkin, Ahmet Demirok

**Affiliations:** ^1^Beyoglu Eye Research and Training Hospital, 34001 Istanbul, Turkey; ^2^Department of Ophthalmology, Gaziantep Sehitkamil State Hospital, Gaziantep, Turkey; ^3^Department of Ophthalmology, Canakkale Onsekiz Mart University, Canakkale, Turkey; ^4^Department of Ophthalmology, Istanbul Medeniyet University Medical School, Istanbul, Turkey

## Abstract

*Purpose.* To evaluate the effect of a fluorometholone/tetrahydrozoline fixed combination on conjunctival graft morphology after primary pterygium excision. *Methods.* The patients who underwent pterygium excision with conjunctival autograft transplantation were randomized into three groups based on postoperative medications as the fluorometholone/tetrahydrozoline group, fluorometholone group, and dexamethasone group. Conjunctival graft thickness was measured with anterior segment optical coherence tomography. The conjunctival graft hyperemia was evaluated using a high definition external camera. *Results.* The mean graft thickness was significantly lower in the fluorometholone/tetrahydrozoline group compared with fluorometholone and dexamethasone groups at 2 weeks (*P* = 0.002 and *P* = 0.012, resp.) and at 1 month after surgery (*P* = 0.003 and *P* = 0.013, resp.). The conjunctival hyperemia score was significantly lower in the fluorometholone/tetrahydrozoline group compared with fluorometholone and dexamethasone groups at 2 weeks (*P* = 0.000 and *P* = 0.000, resp.) and at 1 month (*P* = 0.039 and *P* = 0.040, resp.). The graft thickness and conjunctival hyperemia score were similar among the groups at 1 week and 3 months (*P* > 0.05). *Conclusion.* The findings of the present study revealed that treatment with the fluorometholone/tetrahydrozoline fixed combination may be helpful to decrease graft edema and to achieve better cosmetic appearance at 2 weeks and 1 month after pterygium excision.

## 1. Introduction

Pterygium is a common ocular surface disorder that is characterized by a proliferating fibrovascular tissue that extends onto the cornea. When it causes ocular irritation, disfigurement, or visual impairment caused by its growth over the pupillary axis or induced astigmatism, surgical excision is indicated. Although various surgical procedures have been described for the treatment of pterygium, nowadays the conjunctival autograft transplantation is the most commonly used procedure for the treatment of pterygium [1–4]. This technique has been associated with low complications, low recurrence rates, and also improved favorable cosmetic results. Even though temporary postoperative graft edema after pterygium excision has been reported clinically [4–9], no enough studies have been conducted on the postoperative changes of conjunctival autograft and its associations with postoperative medication.

Usually after pterygium surgery is prescribe antibiotic drop which is used to minimize the risk of infection, a steroid eye drop, used to minimize inflammation, modulate healing, and prevent tissue rejection, and a lubricating drop, used to moisturize and lubricate the eye. The medications analyzed here were fluorometholone acetate ophthalmic suspension that is indicated for use in the treatment of steroid responsive inflammatory conditions of the palpebral and bulbar conjunctiva, cornea, and anterior segment of the eye. Dexamethasone is a potent synthetic member of the glucocorticoid class of steroid drugs and acts as an anti-inflammatory and immunosuppressant. Tetrahydrozoline is an alpha agonist and its main mechanism of action is the constriction of conjunctival blood vessels. This serves to relieve the redness of the eye caused  by  minor  ocular  irritants.

Optical coherence tomography (OCT) is now well recognized as a noncontact technique for high-resolution, cross-sectional tomographic imaging of tissue by measuring optical reflections [10]. With recent advent of new technologies, the utility of OCT in clinical practice has extended to the anterior segment structures of eye such as cornea, conjunctiva, anterior chamber and tear meniscus [11–16]. Visante OCT (Carl-Zeiss Meditec, Dublin, CA, USA) is a commonly used anterior segment OCT (AS-OCT) device for imaging anterior segment structures. It uses a superluminescent light-emitting diode with a wavelength of 1310 nanometers for imaging the anterior segment with a resolution of 18 microns and a penetration of 3 to 4 mm.

This study compares the effect of 3 topical medications, fluorometholone, dexamethasone, and fluorometholone/tetrahydrozoline fixed combination, on conjunctival autograft morphology evaluated with external photography and AS-OCT imaging after pterygium excision.

## 2. Material and Methods

### 2.1. Study Design and Participants

This prospective and interventional study was performed at the Beyoglu Eye Research and Training Hospital. The study was conducted according to the principles of the Declaration of Helsinki and was approved by the local ethics committee. Once the informed consent document has been signed, subjects are considered enrolled in the study.

Forty-five eyes of 45 patients with primary pterygium who underwent pterygium excision with conjunctival autograft transplantation were enrolled in study. Exclusion criteria included prior conjunctival and scleral surgery, a history of a systemic condition that could have compromised wound healing, and a history of any ocular surface disease besides pterygium, uveitis, and retinal vascular diseases. The patients who had a postoperative complication such as hematoma beneath the graft, pterygium recurrence, and pyogenic granuloma were also excluded.

### 2.2. Surgical Procedure

All pterygium surgeries were performed under local anesthesia by the same surgeon (EBO). The head of the pterygium was separated at the limbus and dissected toward the central cornea using a crescent knife. Then, the pterygium and pathologic fibrovascular tissue underlying the pterygium was excised with a blunt and sharp dissection up to the insertion of the medial rectus muscle. Minimal cauterization was used to control bleeding. A free conjunctival autograft of a size equivalent to the recipient bed was harvested from the superotemporal bulbar area with attentive dissection under topical anesthesia to avoid inclusion of any Tenon's tissue. Then, the conjunctival graft was secured in place with Nylone 10-0 sutures. Patients were seen prior to surgery and were thereafter seen at 1 week, 2 weeks, 1 month, and 3 months after surgery.

### 2.3. Study Groups

The 45 patients were subdivided into 3 randomized groups based on postoperative medications. In the fluorometholone group, patients were treated with topical fluorometholone 0.1% (Flarex, Alcon) four times daily and topical Moxifloxacin drop four times daily for a month after surgery. In the dexamethasone group, patients received topical dexamethasone 0.1% (Dexa-sine, Alcon) four times daily and topical Moxifloxacin drop four times daily for a month after surgery. In the fluorometholone/tetrahydrozoline group, patients were treated with topical fluorometholone 0.1% tetrahydrozoline HCl 0.025% fixed combination (Efemoline, Novartis) four times daily and topical Moxifloxacin drop four times daily for a month after surgery. The patients and the clinician who examined the patients were masked in terms of groups.

### 2.4. Visante AS-OCT Measurement

**Figure 1:**
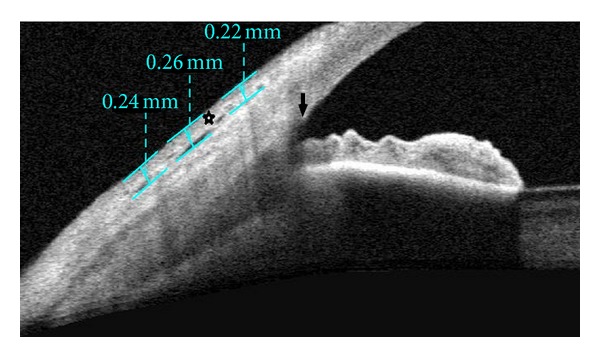
Anterior segment imaging of a patient who underwent pterygium surgery. Conjunctival graft thickness (*asterisk*) at 1, 2, and 3 mm posterior to scleral spur (*arrow*) was measured with a caliper tool.

**Figure 2:**
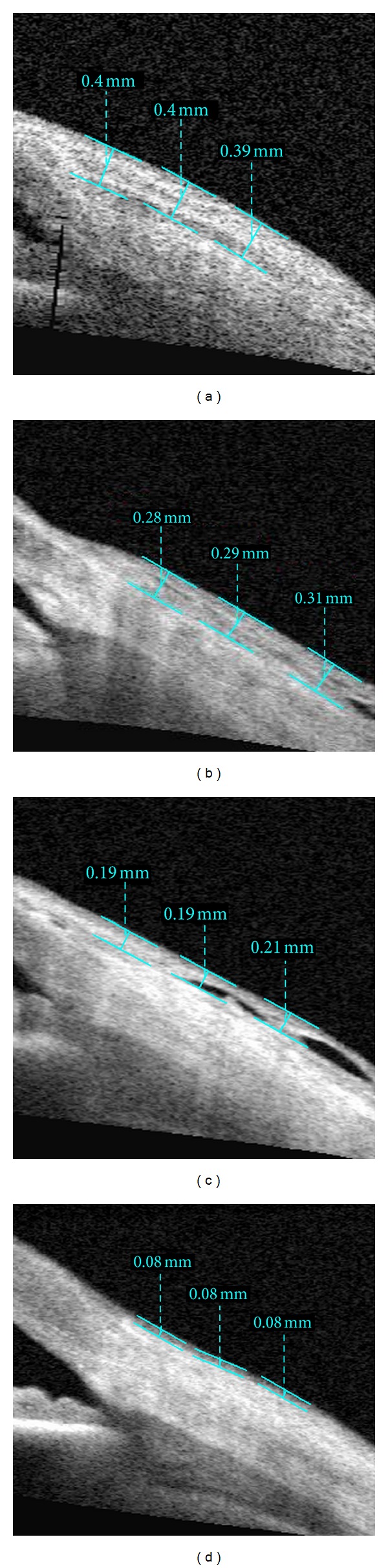
Imaging of AS-OCT shows measurement of thickness of the conjunctival graft after primary pterygium surgery in fluorometholone group at postoperative 1 week (a), 2 weeks (b), 1 month (c), and 3 months (d).

**Figure 3:**
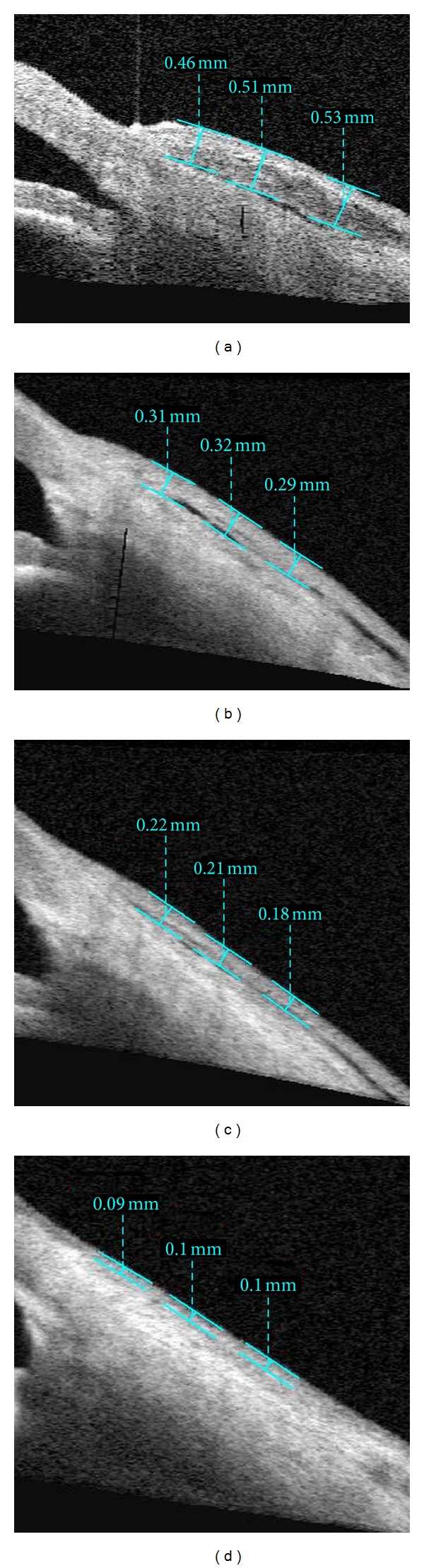
Imaging of AS-OCT shows measurement of thickness of the conjunctival graft after primary pterygium surgery in dexamethasone group at postoperative 1 week (a), 2 weeks (b), 1 month (c), and 3 months (d).

**Figure 4:**
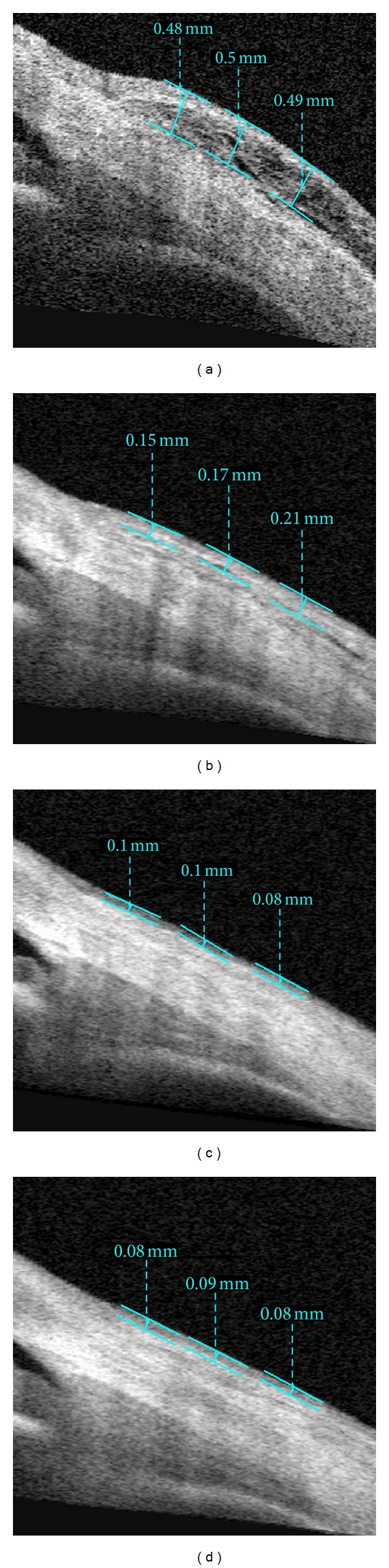
Imaging of AS-OCT shows measurement of thickness of the conjunctival graft after primary pterygium surgery in the fluorometholone/tetrahydrozoline group at postoperative 1 week (a), 2 weeks (b), 1 month (c), and 3 months (d).

Anterior segment measurements using the commercially available Visante OCT (Carl-Zeiss Meditec, Dublin, CA, USA) were performed under the same room conditions by a single clinician who was masked in terms of groups. Imaging with AS-OCT was performed at 1 week, 2 weeks, 1 month, and 3 months after surgery to measure thickness of the conjunctival graft. While the patient's head was kept straight, the imaging was done in the primary position and also the subjects viewed an external fixation target to scan the graft. The AS-OCT measurements were performed at the horizontal meridian in the center of the graft at a line crossing the pupil center. Graft thickness at 1, 2, and 3 mm posterior to scleral spur was measured with a caliper tool provided by the AS-OCT software (Figures 1, 2, 3, and 4) and it was calculated by averaging three measurements. 

### 2.5. Conjunctival Graft Photography

**Figure 5:**
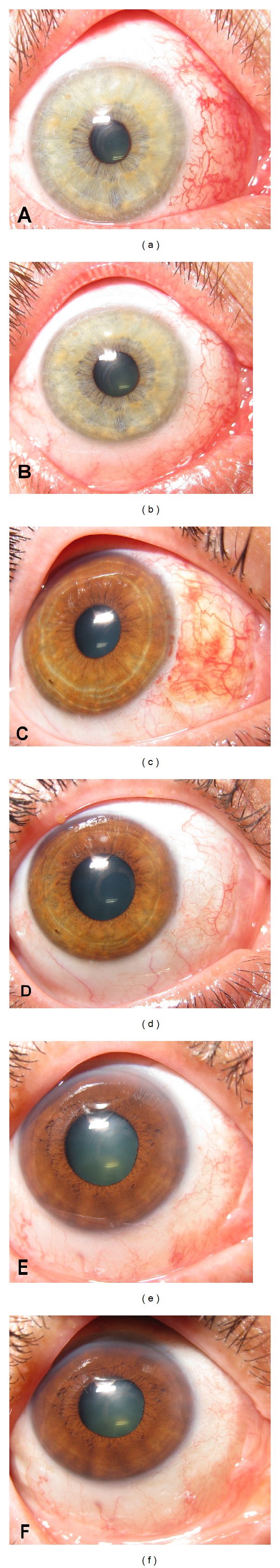
Photographs show area of conjunctival graft implantation after primary pterygium surgery in fluorometholone group at postoperative 2 weeks (a) and 1 month (b), in dexamethasone group at postoperative 2 weeks (c) and 1 month (d), and in the fluorometholone/tetrahydrozoline group at postoperative 2 weeks (e) and 1 month (f).

The conjunctival graft was photographed by a high definition external camera (Figure 5). The conjunctival hyperemia was graded from 0 to 4 using photographs as 0: absence of hyperemia, 1: mild hyperemia, 2: moderate hyperemia, 3: severe hyperemia, and 4: intense hyperemia.

### 2.6. Data Collection

All data were obtained via prospective collection and entered into a standardized computerized database. The data collected included demographic characteristics, clinical examination findings, graft photography, and AS-OCT measurements. The graft photography and AS-OCT measurements were taken by one experienced clinician, and the images were assessed by another ophthalmologist. Both of the clinicians were masked in terms of study groups and measurements time. Patients were seen prior to surgery and thereafter seen at 1 week, 2 weeks, 1 month, and 3 months after surgery.

### 2.7. Main Outcome Measures

Main outcome measures were mean conjunctival thickness and mean graft hyperemia score.

### 2.8. Data Analyses

Statistical analysis was performed using SPSS version 15 (SPSS Inc., Chicago, IL). Fisher exact test was used to compare categorical variables. Paired *t*-tests were performed to compare the graft thickness between various follow-up time points. An ANOVA test was used to compare more than two means. Once a significant difference was found between the means, the Bonferroni test was used for differences. A *P* value of less than 0.05 was considered significant.

## 3. Results

### 3.1. Demographic Profile

This study included 45 eyes of 45 patients. The demographic characteristics of the three groups of subjects are shown in Table 1. No statistically significant differences were observed among the groups in terms of age, gender distributions, laterality, and length of the pterygium (from limbus to apex).

### 3.2. Conjunctival Graft Thickness

**Figure 6:**
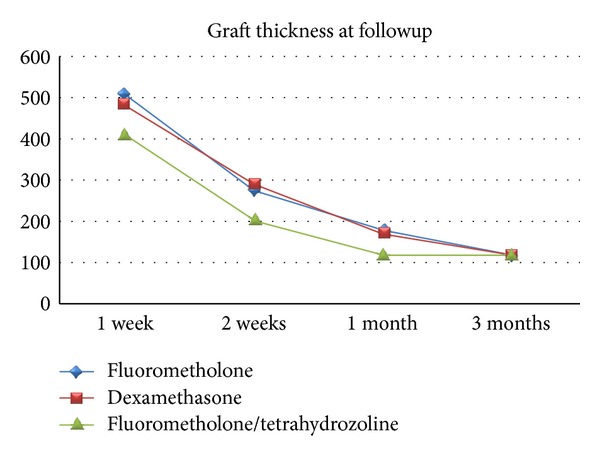
Mean conjunctival graft thickness after primary pterygium surgery.

Table 2 shows the mean conjunctival graft thickness at 1 week, 2 weeks, 1 month, and 3 months after the surgery in three groups. A significant difference was seen at 2 weeks and at 1 month after surgery (*P* < 0.01 and *P* < 0.01, resp.); however, no significant differences were found between the three groups at postoperative 1 week or 3 months (*P* = 0.295 and *P* = 0.891, resp.).  Table 3 shows the pairwise comparisons of three postoperative medications in terms of average graft thickness at different postoperative visits (Figure 6). The mean graft thickness was significantly lower in the fluorometholone/tetrahydrozoline group than in the fluorometholone and dexamethasone groups at 2 weeks (*P* = 0.002 and *P* = 0.012, resp.) and at 1 month after surgery (*P* = 0.003 and *P* = 0.013, resp.).  Table 4 shows a pairwise comparison of mean graft thickness at 1 week, 2 weeks, 1 month, and 3 months after surgery in three groups. Average graft thickness significantly decreased between 1 week and 1 month in all groups. Although the mean graft thickness in the fluorometholone and dexamethasone groups did significantly decrease between 1 month and 3 months, the thickness did not significantly decrease in the fluorometholone/tetrahydrozoline group between 1 month and 3 months.

### 3.3. Graft Hyperemia

Table 5 shows mean graft hyperemia score at 1 week, 2 weeks, 1 month, and 3 months after surgery in three groups. A significant difference was seen at 2 weeks and at 1 month after surgery (*P* < 0.01 and *P* = 0.017, resp.); however no significant differences were found between the three groups at postoperative 1 week or 3 months (*P* = 0.381 and *P* = 0.437, resp.).  Table 6 shows the pairwise comparisons of three postoperative medications in terms of graft hyperemia at different postoperative visits. The mean graft hyperemia score was significantly lower in the fluorometholone/tetrahydrozoline group than in the fluorometholone and dexamethasone groups at 2 weeks (*P* = 0.000 and *P* = 0.000, resp.) and at 1 month after surgery (*P* = 0.039 and *P* = 0.040, resp.).

No other serious or sight-threatening ocular adverse events were observed in any of the 45 patients during any of the follow-up periods.

## 4. Discussion

Currently most research studies into pterygium surgery have focused on recurrence rates, complications of surgery, and adjunctive therapy [17]. From a patient's perspective, the postoperative appearance of the eye is very important. Although conjunctival autograft with or without limbus has been popularized in recent years for closure of the defect, too few studies have mentioned graft morphology and graft appearance after pterygium surgery.

Many previous studies have investigated the causes of conjunctival graft edema after pterygium excision. The exact mechanism of postoperative graft edema remains unknown, but this edema has been attributed to excessive surgical manipulation, the use of Vicryl sutures, graft desiccation, inadequate Tenon's excision, poor graft orientation, young patients, and hematoma of the graft [4, 6, 8]. In addition, a current study by Kheirkhah et al. has conducted a quantitative assessment of conjunctival graft morphology after primary pterygium surgery [18]. They found no significant difference between the 1- and 3-month values after primary pterygium surgery. However there have not been enough studies on the effect of postoperative medication on morphological properties of conjunctival graft.

In this study, we investigated the effect of three medication modalities including fluorometholone, dexamethasone, and fluorometholone/tetrahydrozoline after pterygium surgery where graft thickness was measured by AS-OCT and graft appearance was photographed by an external camera. To our knowledge, this is the first study in this respect. Our results indicate that fluorometholone/tetrahydrozoline fixed combination is more effective for reducing the postoperative graft edema and the graft hyperemia than fluorometholone and dexamethasone during postoperative 1 month.

The fluorometholone/tetrahydrozoline fixed combination consists of a less potent corticosteroid and a decongestant. Fluorometholone helps to relieve inflammation. Tetrahydrozoline 0.05%, a direct acting sympathomimetic or nonselective alpha adrenergic agonist, constricts dilated conjunctival vessels. Interruption of the oculosympathetics can result in autonomic symptoms such as conjunctival injection. Based on the results of this study, we thought that tetrahydrozoline, and not fluorometholone, reduces postoperative graft edema and graft hyperemia. In the literature no studies have been reported regarding the fluorometholone/tetrahydrozoline fixed combination. On the other hand, there are some reports for tetrahydrozoline 0.05%. Osterhoudt and Henretig reported that a sinus node arrest was occurred after ingestion of less than 1 tablespoon of 0.05% tetrahydrozoline-containing eye drops [19]. Lee et al. indicated that tetrahydrozoline use by patients could conceal Horner syndrome [20]. In our study, no serious ocular or systemic side effects were noted during the 3 months of followup.

The study has some limitations. First, this study has a small sample size and larger studies are needed to validate these findings. Further investigation would be required in a larger study population. Second, our study did not include a negative placebo treatment, as the inclusion of a placebo group was thought to be unethical in patients who underwent ocular surgery. Third, the grading of graft hyperemia is subjective and may lead to bias. Fourth, the study did not include a control group of eyes with normal conjunctival thickness, because attempting to determine the entire thickness of normal bulbar conjunctiva, including the epithelium and stroma, is a challenge. Also in our study, graft thickness was easily determined at the early postoperative visits (1 week, 2 weeks, and 1 month), whereas it was more difficult at the late postoperative visit (3 months). 

In summary, this is the first report about the effect of postoperative medication on conjunctival graft thicknesses and conjunctival hyperemia. The findings revealed that the patients treated with fluorometholone/tetrahydrozoline fixed combination had faster graft healing and better cosmetic appearance. However, further study with a larger sample size is required to make a firm conclusion.

## Figures and Tables

**Table 1:** The demographic and clinical characteristics of patients.

	Fluorometholone group	Dexamethasone group	Fluorometholone/tetrahydrozoline group	*P*
Age, year				
Mean ± SD	46 ± 14	50 ± 15	54 ± 15	0.388^a^
Range	28–73	23–72	26–80	
Gender				
Female/male	9/6	5/10	6/9	0.310^b^
Eye				
Right/left	6/9	8/7	10/5	0.343^b^
Length of pterygium, mm				
Mean ± SD	3.8 ± 0.7	3.6 ± 0.9	3.5 ± 0.9	0.781^a^
Range	1.9–5.1	2.1–5.0	1.6–5.2	

SD: standard deviation, ^a^one-way ANOVA test, ^b^Pearson chi-square test.

**Table 2:** Changes of the conjunctival graft thickness after pterygium surgery.

	Fluorometholone group	Dexamethasone group	Fluorometholone/tetrahydrozoline group	*P* ^ a^
	Mean ± SD	Mean ± SD	Mean ± SD	
1 week	509 ± 1777	483 ± 201	412 ± 135	0.295
2 weeks	274 ± 61	290 ± 60	203 ± 43	0.000
1 month	178 ± 59	168 ± 46	118 ± 10	0.001
3 months	111 ± 28	115 ± 30	114 ± 12	0.891

SD: standard deviation, ^a^one-way ANOVA test.

**Table 3:** Pairwise comparison of postoperative medications in terms of mean graft thickness at different postoperative visits.

Groups	1 week	2 weeks	1 month	3 months
Fluorometholone versus dexamethasone	0.687	0.511	0.582	0.996
Fluorometholone versus fluorometholone/tetrahydrozoline	0.133	0.002	0.003	0.499
Dexamethasone versus fluorometholone/tetrahydrozoline	0.267	0.012	0.013	1.0

^a^Paired *t*-test.

**Table 4:** Pairwise comparison of mean graft thickness at different postoperative visits.

Postoperative visits	Fluorometholone group	Dexamethasone group	Fluorometholone/tetrahydrozoline group
1 week versus 2 weeks	0.002	0.009	0.000
2 weeks versus 1 month	0.005	0.000	0.000
1 month versus 3 months	0.003	0.003	0.092

^a^Paired *t*-test.

**Table 5:** Changes of the conjunctival hyperemi after pterygium surgery.

	Fluorometholone group	Dexamethasone group	Fluorometholone/tetrahydrozoline group	*P* ^ a^
	Mean ± SD	Mean ± SD	Mean ± SD	
1 week	3.7 ± 0.4	3.6 ± 0.7	3.4 ± 0.8	0.381
2 weeks	2.8 ± 0.6	3.0 ± 0.7	1.8 ± 0.5	0.000
1 month	1.2 ± 0.9	1.2 ± 0.8	0.4 ± 0.6	0.017
3 months	0.2 ± 0.4	0.3 ± 0.4	0.1 ± 0.3	0.437

SD: standard deviation, ^a^one-way ANOVA test.

**Table 6:** Pairwise comparison of postoperative medications in terms of graft hyperemia at different postoperative visits.

Groups	1 week	2 weeks	1 month	3 months
Fluorometholone versus dexamethasone	1.0	1.0	1.0	1.0
Fluorometholone versus fluorometholone/tetrahydrozoline	0.575	0.000	0.039	1.0
Dexamethasone versus fluorometholone/tetrahydrozoline	0.883	0.000	0.040	0.660

^a^Paired *t* test.
